# The regulators of BCR signaling during B cell activation

**DOI:** 10.1097/BS9.0000000000000026

**Published:** 2019-10-21

**Authors:** Yue Wen, Yukai Jing, Lu Yang, Danqing Kang, Panpan Jiang, Na Li, Jiali Cheng, Jingwen Li, Xingbo Li, Zican Peng, Xizi Sun, Heather Miller, Zhiwei Sui, Quan Gong, Boxu Ren, Wei Yin, Chaohong Liu

**Affiliations:** a Department of Pathogen Biology, School of Basic Medicine, Huazhong University of Science and Technology, Wuhan, China; b Department of Medical Imaging Technology, Clinical and Molecular Immunology Research Center, Medical school of Yangtze University, Jingzhou, Hubei, China; c Department of Intracellular Pathogens, National Institute of Allergy and Infectious Diseases, MT 59840, USA; d Division of Medical and Biological Measurement, National Institute of Metrology, Beijing, China; e Wuhan Children's Hospital, Tongji Medical College, Huazhong University of Science & Technology, Wuhan, China

**Keywords:** BCR, MAPK, PI3K, PLC-γ2, Regulator, Signaling pathways

## Abstract

B lymphocytes produce antibodies under the stimulation of specific antigens, thereby exerting an immune effect. B cells identify antigens by their surface B cell receptor (BCR), which upon stimulation, directs the cell to activate and differentiate into antibody generating plasma cells. Activation of B cells via their BCRs involves signaling pathways that are tightly controlled by various regulators. In this review, we will discuss three major BCR mediated signaling pathways (the PLC-γ2 pathway, PI3K pathway and MAPK pathway) and related regulators, which were roughly divided into positive, negative and mutual-balanced regulators, and the specific regulators of the specific signaling pathway based on regulatory effects.

## 1 INTRODUCTION

The B lymphocyte, or B cell for short, is the antibody producing cell in the immune system. It has three main functions: producing antibodies, presenting antigens, and secreting cytokines involved in immune regulation.

In the bone marrow, B cell development undergoes the stages of progenitor B cell, pre-B cell, immature B cell, and mature B cell. Two major changes in the various stages of B cell development in the bone marrow are rearrangement of immunoglobulin genes and the expression of membrane surface marks.

The pre-B cells are differentiated from pro-B cells and have undergone rearrangement of Ig heavy chain genes. In the cytoplasm of pre-B cells, IgM heavy chain molecules, namely μ chains, can be detected. But due to the absence of light chain (L chain) gene rearrangement, no membrane Ig expression occurs. Instead a surrogate L chain can be combined with the μ chain and act as a surrogate BCR complex on the plasma membrane, and this surrogate BCR takes a significant role in the further development of the pre-B cell. Due to lack of a complete BCR, pre-B cells cannot respond to antigenic stimulation and thus do not have any immune function.^1^

During the immature B cell stage, L chain gene rearrangement occurs, resulting in the formation of completed IgM molecules which express antigen specific receptors on the membrane surface (mIgM). Further differentiation of immature B cells, leads to development to mature B cells that leave the bone marrow to enter immune organs. At this time, IgM and IgD are expressed together on the membrane, and their specificity of antigen recognition are the same.^2^

In the peripheral lymphatic organs, mature B cells are stimulated by antigen, and activated to proliferate and differentiate into short lived plasma cells, which can synthesize and secrete antibodies. Each plasma cell produces only one type of Ig molecule, which has a unique antigen binding specificity. During antibody production, the type of Ig produced is transformed from the IgM isotype to IgG, IgA, or IgE isotypes.^3^

Following antigenic stimulation and B cell activation, some B cells transform into small lymphocytes and stop proliferation as well as differentiation, their IgD disappears, and their life expectancy increases to months or years. When contacting the same antigen again, these B cells quickly become activated and differentiate to plasma cells. Therefore, they are termed “memory B cells,” which play an important role in the secondary immune response.

The BCR is the most numerous surface receptor on the B cell, having 100,000 to 200,000 per cell.^4^ A B cell identifies a pathogen by the homologous BCR on its membrane surface. BCR signaling is essential for normal B cell development and maturation. Mutations in the component genes downstream of the BCR precursor signal block the development of B cells in the bone marrow, leading to antibody defects.^5^ Mutations in BCR co-receptors impair affinity maturation and antibody responses, thereby affecting humoral immune responses^6,7^ Mutations in the TLR signaling component and defects in BAFF-R can also impair B cell development and antigen responses.^1^

Additionally, BCR signaling is also a key pathway for B-cell malignancy. On the one hand, BCR can induce B cell lymphoma by the chronic activation of foreign microorganisms or viral antigens, such as Helicobacter pylori-induced mucosal lymphoid tissue lymphoma and Hepatitis C virus-induced splenic marginal lymphoma.^8^ On the other hand, mutations in related components of the BCR signaling pathway can also induce B-cell lymphomas and autoimmune diseases. About 70% of the newly generated B cells in the body are autoreactive.^9^ The body's self-protection function silences these autoreactive cells through receptor editing,^10^ clone deletion,^11^ and conversion to non-reactive B cells.^12^ Therefore, BCR signaling pathway inhibitors and potent autoimmune checkpoint activators within a short period of time are becoming important targets for the treatment of B-cell malignancies and B-cell dependent autoimmune diseases.^13–15^

Here, we will discuss the BCR mediated signaling pathways and related regulators.

## 2 THE STRUCTURE OF THE BCR COMPLEX

BCR is the specific surface marker of B cells, consisting of an extracellular region, transmembrane (TM) region, and intracellular region. According to the latest research, most of membrane BCRs exist as oligomers rather than individual monomeric receptors. This finding suggests that B cell activation is related to the BCR oligomer opening for binding regulatory molecules to transduce BCR signaling.^4^

The monomeric form of the BCR is made of a membrane-bound form of immunoglobulin (mIg) which is combined with an Igα/Igβ heterodimer non-covalently linked. The mIg of mature B cells is mainly mIgM and mIgD that belong to the immunoglobulin superfamily prototype, which contains the structure of a tetramer chain with two heavy chains (IgH) and two light chains (IgL) connected by a disulfide bond. The intracellular regions of mIg are very short, and the intracellular regions of IgM and IgD contain only three amino acids (KVK). This structural feature prevents mIg from transmitting the signal of antigen stimulation without the participation of other molecules.

Igα and Igβ are also named CD79a and CD79b, which are 47 and 37 kDa glycoproteins, encoded by the *mb-1* and *B29* genes, and belong to the immunoglobulin superfamily.^16^ They both have an Ig domain on the cell membrane surface, an evolutionarily conserved TM region, and a cytoplasmic tail with an immune receptor tyrosine-based activation motif (ITAM), which is about 26 amino acid residues long.^4^The two molecules are covalently bound together through a disulfide bond at the extracellular and TM regions. There are two main functions of Igα and Igβ: to serve as the main signal transduction molecules during antigen binding BCR and to participate in the expression and transposition of mIg.

## 3 BCR-MEDIATED SIGNALING PATHWAY

**Figure 1:**
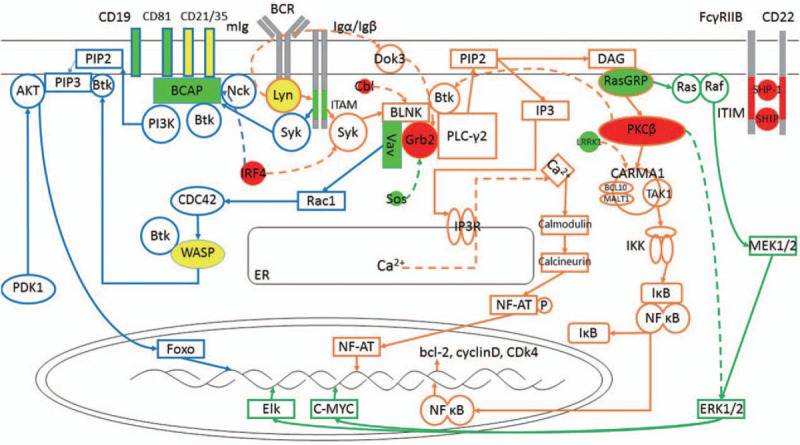
BCR Signaling Pathway. Resting B cells present BCR as a self-inhibiting oligomer. Upon BCR binding to the antigen, actin mediates nanoscale recombination of the receptor clusters, exposing the ITAM domain. Lyn phosphorylates ITAM, recruits Syk and activates downstream pathways. PLC-γ2 pathway: Syk phosphorylates BLNK to form a multimolecular protein complex with PLC-γ2, Grb2 and Btk. Btk and PLC-γ2 are phosphorylated and activate PIP2 to produce second messengers DAG and IP3. IP3 enters the cytoplasm and binds to the IP3R on the ER, resulting in the release of Ca^2+^ from the ER, while the extracellular Ca^2+^ enters the cell under the action of the STIM1 protein. Elevated Ca^2+^ concentrations activate calmodulin and calcineurin, allowing NFAT nuclear translocation and transcriptional activation. DAG on the plasma membrane associates with RasGRP and activates PKCβ, phosphorylates CARMA1 and induces the formation of CBM complexes. The CBM complex recruits TAK1 and IKK and activates the IKK complex to produce a free NF-κB dimer that translocates to nuclear and transcripts targeting genes. PI3K pathway: Both phosphorylated CD19 and BCAP recruit and activate PI3K. The enzyme activity of PI3K is enhanced by Rac1, activation of Rac1 is achieved by Vav, and Vav is recruited to the BLNK-Grb2 complex and phosphorylated. Activation of PI3K results in the phosphorylation of PIP2 to produce PIP3. PIP3 binds Akt and Btk. Akt is phosphorylated by PDK1 and activates FOXO1. Btk can be recruited to the membrane by PI3K, recruiting and activating WASP, which is then phosphorylated by Vav-activated CDC42. MAPK pathway: DAG binds to and activates RasGRP, activates Ras on membrane, GTP binds to Ras to recruit and synthesize Raf, then phosphorylates and activates downstream MAPKK and ERK1/2, and then phosphorylates nuclear transcription factors such as c-Myc. In this figure, green indicates a positive regulator, red indicates a negative regulator, and yellow indicates a bidirectional regulator, the detailed mechanism of which is detailed below (there are many regulators not shown in the figure).

The BCR signal is generated by the binding of a BCR to its cognate antigen. The majority of BCR complexes on resting B cells exist as self-inhibiting oligomers. Following BCR binding with antigen, there is actin mediated nanoscale recombination of receptor clusters, opening BCR oligomers to reveal the ITAM domains. This allows for intracellular signal transduction, as the two tyrosines of the ITAM are then phosphorylated by Src-family kinases, such as Lyn, and provide sites for recruitment and activation of Syk. This leads to the formation of a BCR/Syk complex and activation of several BCR controlled signaling pathways^4^ (Fig. 1).

### 3.1 The PLC-γ2 pathway

In the PLC-γ2 pathway, the B cell linker (BLNK) is a key adaptor protein that recruits Syk and Btk. After BCR antigen stimulation, Y204, an evolutionarily conserved non-ITAM tyrosine residue, near the ITAM of Igα recruits BLNK through SH2 domain binding to phosphorylated Igα. Syk is grouped to phosphorylated ITAM of Igα/Igβ, then activated, and phosphorylates BLNK, which combines with PLC-γ2, Grb2 and Btk to form a multimolecular protein complex.^17^ Transphosphorylation of Syk and Lyn fully activates Btk. Btk interacting with phosphatidylinositol-4-phosphate 5-kinase (PIP5K) results in increased synthesis of PIP2.^18^ Syk and Btk activate PLC-γ2 via tyrosine phosphorylation, and catalyzes PIP2 to second messengers DAG and IP3.

In the first stage of Ca^2+^ release, IP3 binds to its receptor IP3R, a ligand-gated Ca^2+^ channel on the ER membrane, resulting in an increase in Ca^2+^ release from the ER. Due to the small number of Ca^2+^ in the endoplasmic reticulum, its reduction is immediately detected by the STIM1 protein which becomes separated from Ca^2+^ and accumulates in ER regions related to the cell membrane. STIM1 then combines with Orai channels and activates Ca^2+^ release-activated Ca^2+^channels (CRAC) in the cell membrane directly, allowing the extracellular Ca^2+^ to enter the cytosol. This second stage of Ca^2+^ transference is known as store-operated Ca^2+^ entry (SOCE), which maintains a continually increasing level of the intracellular Ca^2+^, and is necessary for continued Ca^2+^ signaling.^18^

Elevated Ca^2+^ concentrations activate Ca^2+^ dependent protein, calmodulin. Calmodulin activates calcineurin, which dephosphorylates NFAT, resulting in nuclear translocation and transcriptional activation of nuclear factor of activated T cell (NFAT). Thus, Ca^2+^ concentrations regulate NFAT nuclear localization, since any decrease in Ca^2+^ will deactivate calmodulin, resulting in phosphorylation of NFAT and exiting the nucleus.^19^

Second messenger DAG is confined to the plasma membrane and connects to RasGRP, this mediates PKCβ activation, which can phosphorylate CARMA1 and induce the conformational change of CARMA1 to combine with BCL10-MALT1 and become CARMA1-BCL10-MALT1 complex (CBM).^20^ TAK1 and IKK are recruited to phosphorylated CARMA1 through CBM. In resting B cells, the NF-κB dimer is stored in the cytoplasm and binds to IκB protein, but when TAK1 contacts IKK, TAK1 phosphorylates IKK, then activates the IκB kinase (IKK) complex, resulting in IκB phosphorylation and proteasomal degradation. This free NF-κB dimer (p50/cRel) translocates into the nucleus to transcribe targeted genes, which leads to expression of BCL-2, cyclin D, CDK4 and other important molecules. In addition, PKCβ activates MAPK family members to induce cell survival as well as proliferation.^21^

In the PLC-γ2 pathway, if PLC-γ2 lacks the autoinhibitory C-terminal Src homology 2 (cSH2) domain, PLC-γ2 associated antibody deficiency and immune dysregulation (PLAID) will occur.^22^ The possible reason is that the cSH2 structure of PLC-γ2 can stabilize the early signaling complex of BCR cross-linking. If it is lacking, it will lead to the imbalance of B cell surface actin polymerization, which will reduce the BCR recruitment, insufficient phosphorylation of Syk, Btk, and BLNK, and the co-localization of the BCR signaling complex is reduced and the Ca^2+^ response is insufficient. The inhibitory factor Cbl of Ca^2+^ signaling is co-localized with BCR, which further inhibits the activity of PLC-γ2.^23^ At the same time, impaired Ca^2+^ signaling can also cause various immunodeficiency-related diseases, such as X-linked gamma globulinemia (XLA)^24^ and common variant immunodeficiency (CVID).^25^

### 3.2 The PI3K pathway

In B cells, PI3Kδ plays a role in signals from BCR, CXCR4, CXCR5, BAFF, and CD19. It is made up of the regulatory subunit p85 and the catalytic subunit p110δ. Regulatory subunit p85 includes an SH2 domain that recruits PI3K to tyrosine kinase linked receptors and their substrates.^26^

Both phosphorylated CD19 and BCAP recruit and activate PI3K. Following BCR stimulation by antigen, Lyn phosphorylates the SH2 domain in the p85α subunit of PI3K that is located on the intracellular domain of the BCR regulatory membrane receptor, CD19. BCAP, an adaptor molecule of PI3K, is coupled to the BCR by the adaptor protein Nck. After coupling with the BCR, Syk as well as Btk phosphorylate BCAP and then BCAP binds with PI3K.

The enzymatic activity of PI3K is enhanced by the GTPase, Rac1. Activation of Rac1(Rac1-GTP) is through the guanine nucleotide exchange factor Vav, which is recruited to the BLNK-Grb2 complex and then phosphorylated.

P110 subunit of PI3K activation results in phosphorylation of membrane PIP2 to generate PIP3. Akt and Btk bind through their PH domains to PIP3. Akt is recruited to the cell membrane by binding to PIP3, here it is phosphorylated by 3-phosphoinositide-dependent kinase-1 (PDK1) and enters the cytoplasm to activate transcription factors involved in cell survival such as FOXO1. BTK recruitment to the cell membrane through PIP3 interaction results in recruitment and activation of Wiskott Aldrich Syndrome protein (WASP), an actin nucleation promoting factor. WASP binds BTK through its SH3 domain and is then phosphorylated by CDC42, which was activated by Vav.

Overactivation of the PI3K pathway leads to phosphoinositide 3–kinase d syndrome (APDS), a type of immunodeficiency disease with major clinical symptoms including recurrent sinus infection, conjunctivitis, bacterial lymphadenitis and herpes virus infection, and benign lymphocyte proliferation, malignant tumors and autoimmune manifestations. Insufficient activation of the PI3K pathway also causes immunodeficiency, which usually manifests as infections in the lungs and intestines. These patients have a low number of B cells and exhibit panhypogammaglobulinemia or agammaglobulinemia from an early age.^27^

### 3.3 The MAPK pathway

Ras GTPase activation, which in turn activates the Ras-Raf-MEK-ERK pathway, is the most clearly discussed MAPK cascade. In B cells stimulated by antigens, DAGs bind and activate RasGRP, which activates the molecular switch protein Ras which is fixed on the cell membrane, transforming it from Ras-GDP to Ras-GTP state. GTP bound Ras recruits Raf to the cell membrane. The membrane localized Raf protein is activated by enzymatic transphosphorylation through forming dimers, which then phosphorylates and activates MAPKK, MEK1, and MEK2, the specific MAPKKs in the ERK cascade. ERK1 and ERK2 are activated by MEK-dependent dual phosphorylation in the participation of enzymes, transmitting the signal of MEK1 and MEK2. The activated ERK is transported to the nucleus. ERKs phosphorylate nuclear transcription factors, c-Myc, Myb, Elk, and downstream kinases, RSKs, MNKs, MSKs, to further spread the signal and increase the number of ERK cascade regulated targets.^28^

CARD protein–BCL-10–MALT1 (CBM) signalosomes plays an important role in the MAPK pathway and mediates B/T cell proliferation and differentiation, metabolic reprogramming and survival after antigen recognition. Its genetic alterations also cause a variety of immunodeficiencies, autoimmune diseases and lymphomas. For example, in MALT lymphoma, somatic gain-of-function (GOF) mutations of CARD11, BCL-10, and MALT1 cause overexpression of BCL-10 or MALT1, causing abnormal NF-κB activation and formation of malignant tumors.^29^ At the same time, loss-of-function (LOF) variants can also occur in children with combinatorial immunodeficiency induced by components used in CBM.^30^

## 4 THE REGULATORS OF BCR SIGNALLING

### 4.1 Integrated Regulators

#### 4.1.1 Positive Regulators

BCAP is a positive regulator of BCR signaling. In the PI3K pathway, BCAP associates with PI3K and regulates its localization.^31^ In the PLC-γ2 pathway, BCAP contains 3 proline-rich sequences that serve as binding sites for PLC-γ2. The cooperation of BCAP and BLNK recruits PLC-γ2 for its activation in membrane rafts. The lack of BCAP in B cells reduces PLC-γ2 activation and Ca^2+^ flux. In addition, BCAP also regulates the expression of c-Rel. BCAP deficiency decreases c-Rel expression and the effector molecules CDK4, Bcl-2 and cyclin D, which are for B cell survival and proliferation.^32^

BCR-induced signaling can be affected by coregulatory molecules.^33^ For instance, CD19 is a positive regulator of BCR signaling.^34^ BCR cross-linking with CD19 reduces the BCR activation threshold by reducing the internalization of BCR, thus extending the residence of BCR in lipid rafts.^35^The co-receptor CD21/35 on the surface of B cells maintains B cell self-tolerance by regulating the turnover rate of CD19 on the cell membrane, inducing internalization of CD19 and inhibiting Ca^2+^ release.^36^ CD21/35 can also inhibit BCR signaling by isolating CD19 from BCRs.^37^ Moreover, the co-receptor CD81 may promote BCR signaling by linking and immobilizing CD19.^38^

CD40 and BAFF-R are positive regulators of BCR signaling. Both belong to the tumor necrosis factor receptor (TNFR) family. In the NF-κB pathway downstream of PLC-γ2, clAP1/2 is an E3 ubiquitin ligase group recruited by tumor necrosis factor receptor-associated factor 2 (TRAF2). CD40 or BAFF-R can prevent NIK from being ubiquitinated and degraded by recruiting TRAF3, which binds to the TRAF2-clAP1/2 complex. When TRAF3 is present, clAP1/2 is activated and ubiquitinates TRAF3 for degradation, while NIK is spared and continues to bind to the downstream kinase IKK to ensure that the NF-κB pathway is not constitutively inhibited.^39,40^ In the PI3K pathway and MAPK pathway, CD40 and BAFF-R promote BCR downstream signaling through similar mechanisms.^41–45^Conversely, non-receptor tyrosine phosphatase PTP1B and hematopoietic protein tyrosine phosphatase inhibit this pathway to down-regulate BCR signaling.^46,47^

Reactive oxygen intermediates (ROI), such as H2O2 are positive regulators of BCR signaling. Crosslinked BCRs lead to Ca^2+^-dependent ROI generation.^48^ In the catalytically active sites of tyrosine phosphatases (e.g., CD45 and SHP-1), it is possible for H2O2 to oxidize conserved cysteine residues by a plasma membrane-associated NADPH oxidase so that it does not active.^49^ Thus, BCR proximal phosphatases are inactivated, facilitating BCR-induced signal transduction.^33^

Recent studies have shown that there is an interaction between microRNA and BCR signaling.^50^ MicroRNA can affect B cell proliferation, apoptosis, and sensitivity to BCR stimulation by regulating B cell transcriptional gene expression, thereby regulating BCR signaling.^51^ Conversely, BCR signaling can affect the expression of certain microRNAs. Among them, the relatively clear miR-155 is considered to be a positive regulator of BCR signaling. It can upregulate BCR signaling by targeting the up-regulation of transcription factor PU.1, down-regulating the expression of SHIP-1, and increasing the sensitivity of B cells to BCR stimulation.^52^ Other micro-RNA regulation of BCR signaling are still under study.

Additionally, new signaling molecules are being discovered to enhance positive BCR signaling. Leucine-rich repeat kinase 1 (LRRK1) is a member of the ROCO family of proteins that cooperates with CARMA1 to regulate NF-κB activation and BCR-induced B cell proliferation.^53^

#### 4.1.2 Negative regulators

The Cbl protein is a negative regulator of BCR signaling. In the PLC-γ2 pathway, Cbl competitively inhibits PLC-γ2 through binding of its SH2 domain to phosphorylated BLNK, resulting in the inhibition of PLC-γ2 phosphorylation.^54^ In the Ras pathway, Grb2 recruits Sos to the membrane, resulting in activated Ras.^55^ The competitive binding of Cbl and Sos to Grb2 is exclusive mutually because their proline-rich regions compete for the same binding site in the Grb2 SH3 domain.^56^ Therefore, Cbl negatively regulates the Ras pathway through prohibiting Sos recruitment to the membrane.

PKCβ is a negative regulator of BCR signaling. PKCβ, which is a feedback inhibitor of Btk activation, inhibits BCR signaling and Ca^2+^ mobilization by phosphorylating a short stretch of highly conserved S180 serine residues in the TH domain of Btk.^17^

Growth factor receptor binding protein 2 (Grb2) is a negative regulator of Ca^2+^ signaling downstream of BCR signaling.^57^ Lyn phosphorylates the adaptor downstream of tyrosine kinase 3 (Dok3) after BCR cross-linking, allowing the SH2 domain of Grb2 to interact with Dok3. Then, they translocate to PIP3 regions in the cell membrane through the Dok3 PH domain, inhibiting Btk activation by competitively binding to PIP3, thereby inhibiting Btk-dependent PLC-γ2 phosphorylation.^18^ In addition, Grb2 also interacts with SHIP, which binds to CD22 and FcγRIIB1, suggesting that Grb2 may integrate a coreceptor that inhibits BCR-mediated Ca^2+^ mobilization.^57^

The transcription factor interferon regulatory factor 4 (IRF4) is a negative regulator of BCR signaling.^58^ Possible mechanisms are:

1. inhibiting BCR-induced calcium signaling by increasing expression of SHIP^59,60^;

2. increasing phosphorylation of Syk and BLNK by decreasing BCAP expression and decreasing PI3K/Akt pathway activity;

3. promoting actin polymerization by inhibiting the interaction between BCR and co-receptor CD19, increasing the BCR signaling threshold, and down-regulating BCR signaling.^38,61^

#### 4.1.3 Bidirectional regulators

The actin cytoskeleton of B cells is a bidirectional regulator of BCR signaling.^62^ Its main component is the cortical actin network. The cortico-actin network is part of the actin cytoskeleton made of actin filaments that are linked by actin-binding proteins and are bound to transmembrane proteins with the help of ezrin/radixin/moesin (ERM) proteins.^63,64^ F-actin is anchored to the plasma membrane through ERM proteins to form distinct compartments on the membrane that limit the lateral movement of membrane proteins. The cytoplasmic domain of ERM proteins extend into the cortical actin network. Thus, a cortical actin network can inhibit BCR aggregation by limiting BCR lateral flow. To initiate BCR signaling, the cortical actin network must first be disassociated. On one hand, the actin cytoskeleton positively regulates BCR signaling. There are three possible mechanisms:

1. B-cell proliferation is triggered by actin recombination, and the area of contact between B cells and antigen on the membrane is increased, thereby increasing the number of antigens that interact with BCRs.^65^
2. Amplify the BCR signal by enhancing the formation of BCR microclusters.^66,67^
3. Act as a scaffold for BCRs to recruit relevant signaling molecules, therefore promoting BCR signaling.^68–71^

On the other hand, the actin cytoskeleton also negatively regulates BCR signaling. The possible mechanisms are:

1. Activated BCRs induce actin polymerization for B cell spreading and accumulation of BCR signaling microclusters. Approximately 5 min into these events, SHIP-1 is activated to reduce the F-actin within the BCR microclusters,^65,66,72,73^ resulting in BCRs collapsing into a central cluster, and also contraction of the B cell. This halts BCR signaling and decreases the signaling,^66^ which is crucial for maintaining B cell tolerance.
2. After BCR activation, recruitment and phosphorylation of actin binding protein (Abp-1) can be induced.^74,75^ Abp-1 recruits actin to clathrin-coated pits and creates forces on the neck of dynamin to help the vesicles detach from the plasma membrane, thereby accelerating antigen uptake and BCR internalization and down-regulating BCR signaling.^75–79^
3. Abp-1 also promotes BCR microcluster polymerization and B cell contraction and recruits inhibitory molecules SHIP-1 and HPK1 to BCR microclusters to attenuate BCR signaling.^80^

Hematopoietic progenitor kinase 1 (HPK1) can inhibit BCR signaling by promoting phosphorylation and ubiquitination of BLNK.^81^

WASP and N-WASP are a pair of bidirectional regulators for BCR signals. Wiskott–Aldrich Syndrome Protein (WASP) is a cytoskeleton-regulatory protein unique to hematopoietic cells, and N-WASP has 50% homology with it. After activation of BCRs, Btk is activated and stimulates PIP2 production by activating Vav, the guanine nucleotide exchange factor of Cdc42, thereby inducing WASP to be phosphorylated and activated,^76^ translocating it to the cell surface. Activated WASP stimulates actin polymerization through binding to Arp2/3,^82–84^ promoting lateral migration of BCRs and B cell proliferation.^61,65,72,85^ At this point, BCR signaling reaches a maximum level and subsequently, SHIP-1 is activated to dephosphorylate PIP3, the binding site of Btk on the plasma membrane,^86^ thereby inhibiting WASP activity and in turn activating N-WASP.^66^ This mechanism may also be related to the signal transducing protein, Grb2.^87–90^ Activated N-WASP promotes the reorganization of F-actin and internalization of BCRs by vesicles,^91–93^ inducing the conversion of B cells from diffusion to contraction, and causing BCR microclusters to accumulate into a central cluster. Thus, the BCR signaling is down-regulated, and B-cell self-tolerance is enhanced.^94^

The complement system and Toll-like receptors are bidirectional regulators of BCR signaling and play an important role in B cell self-tolerance.^95^

In B cells the complement system includes complement proteins and complement receptors, and the combination of them can trigger the complement cascade, which in turn regulates BCR signaling. The common complement protein is C3, and common complement receptors include C3a receptors that bind to C3a, the complement receptor type 1 (CR1, CD35) that binds to C3b and C4b, and complement receptor type 2 (CR2, CD21) that bind to C3d and CR3 (CD11b/CD18), as well as CR4 (CD11c/CD18) that bind to iC3. In human B cells, CR1 and CR2 have mutual antagonistic effects.^96–98^

The major TLRs in human B cells are TLR1/2, TLR6, TLR7, TLR9, and TLR10. When BCR or CD40 is activated, TLR expression is increased,^99,100^ so the TLR content in plasma cells and memory B cells is higher than naive B cells, which makes them more sensitive to antigenic stimulation.^101^ The TLR on the plasma membrane and the TLR on the endosome has an antagonistic effect. Existing studies have shown that TLR9 on the plasma membrane has an inhibitory effect on endosomal TLR9. When BCR activates and produces a signal response in response to endosomal TLR9, the plasma membrane TLR9 can inhibit and down-regulate BCR signaling through binding to different ligands, thereby increasing B cell tolerance.

When cells are damaged or stimulated by self-molecules (such as LPS, nucleic acids, zymosan, etc), the B-cell complement system and TLR signal are activated, and there exists a combination of TLR and CR and BCR. Studies have shown that cross-linking of CR1 with TLR9 can down-regulate BCR signaling.^95^ In addition, under the conditions of autoimmune disease, both the complement system and TRL can enhance the recognition rate of B cells against autoantigens, thereby improving the tolerance of B cells.^102–106^

Transmembrane tyrosine phosphatase, CD45, and tyrosine-protein kinase, Csk, are a pair of antagonistic regulators that regulate Lyn activation. CD45 promotes Lyn activation, whereas Csk inhibits its activation. Lyn receptor-induced tyrosine phosphorylation increases significantly with reduction in Csk and decreases significantly with reduction in CD45. BCR signaling is balanced by CD45 and Csk activity, thereby further regulating the Src-PTKs carboxy-terminal tyrosine phosphorylated state.^55^

### 4.2 Regulators of ITAM and ITIM

There are many membrane molecules on the B cell membrane, such as FcγRIIB, FCRL, CD22, Siglec-10, CD31, mouse PIR-B/human LIRB1 and LIRB2PD-1, BTLA, CD5, and CD72.^107,108^ Some of the domains have a tyrosine-based activation motif (ITAM) and others have a tyrosine-based inhibition motif (ITIM). When the antigen-antibody complex binds to them, Src family kinases phosphorylate the tyrosines on ITIM or ITAM, thereby mediating binding to its downstream SH2 domain containing molecule, including Igα/β heterodimers, Lyn, Syk, Slp65, CD19, Btk, BLNK, and Vav1, and promote or suppress corresponding BCR signaling.^107,109^

Among them, FCRL1 contains two ITAM motifs, which can up-regulate BCR signaling by enhancing BCR-induced Ca^2+^ mobilization and proliferation.^110^ FCRL2-5 has an ITIM motif that inhibits Ca^2+^ mobilization and down-regulates BCR signaling by recruiting and activating SHP-1which has SH2 domains.^111–115^ SHP-1 inhibits the phosphorylation of Syk, thereby inhibiting the BCR signaling pathway.^116^

Transmembrane protein CD22 is a negative regulator of BCR signaling. Random activation of Src kinases related to BCR activation phosphorylates the tyrosines of the CD22 ITIM. These phosphotyrosines recruit SHP-1 through their SH2 domain.^33^ SHP-1 inhibits BCR-induced signaling by removing tyrosine phosphorylation of signaling molecules mentioned above.^117^ Paired immunoglobulin receptor B (PIR-B) and Siglec-G negatively regulate BCR signaling by the same mechanism. In addition, CD22 interacts physically with PMCA4, causing plasma membrane calcium-ATPase 4 (PMCA4) to weaken the BCR signaling in a SHP-1-dependent manner by excreting Ca^2+^ from the cytosol.^118^

FcγRIIB is a low affinity Fc receptor of IgG. Phosphorylation of the ITIM within the FcγRIIB cytoplasmic domain also inhibits BCR signaling. Once Lyn phosphorylates FcγRIIB, FcγRIIB recruits SHP-1^119,120^ and SHIP.^121^ Inositol 5-phosphatase 1 (SHIP1), which contains a SH2 domain, catalyzes the dephosphorylation of PIP3, inhibiting the PH domain-dependent recruitment of BTK to the membrane. This eliminates PLC-γ2 activation and Ca^2+^ flux, thus inhibiting signals produced by antigen bound to BCR.^86^ Additionally, phosphatase and tensin homologues (PTEN) negatively regulate BCR signaling through the same mechanism.^17^

SH2-domain-containing protein tyrosine phosphatase2 (SHP-2) acts synergistically with FcγRIIB to dephosphorylate the adaptor protein Gab1, thereby blocking the PI3K pathway.^122^

Also, PTPN22 inhibits the activity of Syk, PLC-γ2 and Akt and thus inhibits the BCR signal through the PI3K pathway.^123^

Vav is the positive regulator of the PI3K pathway. The Vav family proteins (Vav1, Vav2, Vav3) are guanine nucleotide exchange factors (GEFs) in cytoplasm of the GTPases of the Rho family and are essential for BCR-induced Ca^2+^ influx.^124^ Binding of Vav to Grb2 and BLNK results in potent Rac1 activation following BCR stimulation. Rac1-GTP, in turn, activates PI3K.^125^ Furthermore, Grb2 and BLNK localize Vav to lipid rafts to promote the most efficient activation of B cells.^126^ Through this mechanism, Vav can promote the continuous production of PIP3 and calcium influx, positively regulating the response of B cells.^127^

The Scr family PTK, Lyn, is a bidirectional regulator of BCR signaling. Activation of Lyn results in the phosphorylation of ITIM, and the decrease of Lyn reduces the phosphorylation of ITIM, thereby inhibiting the suppression signals of FcγRIIB and CD22, causing BCR hyper-responsiveness.^55^

### 4.3 Regulators of SOCE pathway

In the SOCE pathway, the oligomerization and assemblage of STIM1-Orai are regulated by many regulating proteins.

When the cytoplasmic Ca^2+^ concentration is low, CRACR2A connects to STIM1 and Orai1, forming a ternary complex in the cytoplasm that stabilizes the CRAC active complex.^128^ After the rise of cytoplasmic Ca^2+^, CRACR2A can unbind STIM1 and Orai1 and inhibit the influx of extracellular Ca^2+^, thus regulating Ca^2+^ mobilization bidirectionally.^18^

The Ca^2+^-binding integrated ER membrane protein junctate, a connecting protein between STIM1 and Orai1,^129^ mediates the transport of STIM1 clusters at the ER-PM junction and promotes Ca^2+^ influx.^130^

LPA5 is a receptor for lysophosphatidic acid (LPA) and may inhibit BCR signaling by inhibiting the activity of IP3R to down-regulate Ca^2+^ release, and subsequently inhibiting expression of CD69 and CD86 under antigen-specific induction.^131^

Septin, one of the GTP binding proteins, is involved in the organization of membrane microdomains of STIM1 and Orai1 by forming hetero-oligomeric complexes.^132,133^ Septin repositions in plasma membrane during ER Ca^2+^ storage depletion, promoting binding of STIM1 to ER-PM junction and recruitment of Orai1.

Surfeit locus protein 4 (Surf4) can bind to STIM1 then interact with the ER region of STIM1, this inhibits the transport of STIM1 to the junction of SOCE and ER-PM, thus inhibiting the SOCE pathway.

Moreover, inositol 1,4,5-triphosphate 3-kinase B (Itpkb) is also a negative regulator of the SOCE pathway which inhibits SOCE by phosphorylating IP3 to form IP4.^18^

### 4.4 Regulators of MAPK pathway

In the Ras-MAPK pathway, GEFs, such as RasGRP, RasGRF, and SOS, are important positive regulators of Ras that control and induce the transference between the GDP-binding state to activated GTP-bound state. The conversion is moved backward by GTPase-activated proteins (GAPs), for example NF1 and RasGAP, which act as negative regulators, thereby promoting Ras's inner GTPase activity, leading to the transformation from GTP to GDP. Therefore, the Ras activation circulation is controlled by the balance of GEFs and GAP activities.^134^

In addition, the regulation mechanisms of ERK in mature B cells is via the negative regulation of DAG signaling mediated by phosphorylation of DAG by DAG kinase (DGK) and conversion to the phosphatidic acid (PA), thus inhibiting the activity of ERK in mature follicular B cells.^28^

## 5 BCR SIGNALING IN CSR

The naïve B cells that secrete IgM are the main body of the primary immune response and, when activated by antigenic stimulation, rapidly proliferate and form a germinal center (GC) with the help of follicular helper T cells (Tfh).^135^ In GC, B cells continue to proliferate and undergo somatic hypermutation (SHM) and in IgH constant region, Cμ is replaced by Cγ, Cα or Cε, resulting in class switched recombination (CSR)^3^ in the immunoglobulin V region under the action of activation-induced cytosine deaminase (AID) to differentiate into memory B cells or plasma cells.^136,137^ CSR is achieved by excision and reconnection of DNA. By recombining the DNA sequence between the two switch (S) regions, the VDJ fragment of the rearrangement completed gene is placed upstream of a new constant region fragment. Thereby, an Ig having the same variable region and different isotype of the constant region is generated.^138^ Among them, low-affinity GC cells with broadly reactive BCRs differentiate into memory B cells, which is beneficial to increase the breadth of antibody responses, whereas high-affinity and intermediate-affinity GC B cells tend to differentiate into plasma cells and continue to maintain GC cells.^139^

Different types of memory B cells secrete different immunoglobulins, including IgM, IgD, IgG, IgE, and IgA, and they migrate to corresponding sites with the help of chemokine receptors (e.g., CXCR3, CXCR4, CXCR5, CXCR6, etc) to exert an immune function. Among them, the signal pathways of IgM and IgD are similar.^2^

Memory B cells expressing IgG and IgE are more responsive to BCR signaling.^140^ Because the cytoplasmic regions of mlgG and mIgE contain immunoglobulin tail tyrosine (ITT), ITT can be phosphorylated by Syk,^131^ which can provide binding sites for growth factor receptor binding protein 2 (Grb2) via the SH2 domain then enhance the phosphorylation of BCR downstream effector proteins and enhance Ca2+ mobilization.^140^ In mIgM-expressing B cells, Grb2 is a negative regulator that interacts with the inhibitory surface receptor CD22, cytoplasmic adaptor proteins Dok3 and SHIP, and inhibits BCR signaling.^141–143^ However, in B cells expressing mIgG and mIgE, the binding of Grb2 to CD22 or Dok3 is blocked by the binding of ITT with Grb2, thereby blocking the negative regulatory pathway. On the other hand, Grb2 interacts with Btk to promote phosphorylation of PLC-γ2 by Btk, thereby reducing the activation threshold of mIgG- and mIgE-containing memory B cells.^131^ In addition, IgG enhances BCR oligomerization after antigen stimulation, promoting Syk recruitment and Ca^2+^ mobilization.^144^

IgE is an antibody involved in allergic reactions. Its expression on the cell surface is much lower than that of BCR containing mIgG and mIgM. By reducing the survival life of IgE-type B cells, the body suppresses the excessive production of IgE, thereby reducing B-cell self-reactions and allergic reactions.^145^

IgA mainly exists on the exposed skin surface and mucosal secretions and belongs to the body's first line of defense.^146^ Since mIgA does not contain ITT, the BCR signal is not enhanced.^140^

AID is a B cell-specific factor that is specifically expressed in the GC of peripheral lymphoid organs after antigen stimulation.^147^ AID can be used to decarboxylate cytidine into uridine in the immunoglobulin V region, causing base mismatches and excision repair. In addition, DNA double-strand breaks (DSBs) are also generated between two S regions in the heavy chain gene cluster, resulting in rearrangement of the CH locus as well as loss of the insertion sequence,^148,149^ and rejoining two fracture areas by non-homologous end joining (NHEJ), CSR was completed.^150,151^ Under the action of LPS and IL-4, PTEN expression was reduced, AKT and FOXO1 were hyperphosphorylated, inhibited AID expression in GC of IgM-BCR, and at the same time downregulated the chemokine receptor CXCR4, thereby reducing the conversion to IgG class.^152,153^ By integrating signals from the non-classical and classical NF-κB pathways, BCRs and TLRs synergistically induce AID and T cell independence.^154^ TLR ligands, such as LPS and CpG, can promote T cell-dependent specific antibody responses and have been used extensively as vaccine adjuvants.^155–157^ CD38,^158^ CD40,^159^ CD180,^160^ TACI,^161^ and IL-10R^162^ can enhance TLR-dependent CSR. In addition, in the differentiation of memory B cells and plasma cells, IRF4 can promote GC formation and plasma cell differentiation,^163^ MITF is a corresponding antagonist factor.^164^ B lymphocyte induced mature protein 1 (Blimp-1) can promote plasma cell differentiation,^136^ and BTB and CNC homolog 2 (Bach2) is the corresponding antagonist factor.^165^

## 6 CONCLUDING REMARK

BCR signaling is a series of signals generated by B cells under the stimulation of antigens. It stimulates the differentiation of B cells to produce an immune response, helping the body to fight against pathogens. However, excessively strong BCR signal responses can make B cells highly reactive, which is the cause of autoimmune diseases. In addition, an abnormal increase in BCR signaling can also lead to B-lymphocyte malignancy. Under normal conditions, when the BCR is activated by antigenic stimulation, B cells will start up their own inhibitory pathway to down-regulate BCR signaling, which is the basis of B cell tolerance. Among them, in the BCR signaling pathway, there are many phosphorylation of receptors and key molecules, so BCR signaling can be down-regulated by dephosphorylation. In the downstream NF-κB pathway, there are many key molecules that require ubiquitination, so deubiquitinating enzymes (DUB), such as CYLD, A20, OTUD7B, etc, can also down-regulate BCR signaling by deubiquitinating related molecules.^166–169^ In contrast, signal molecules that originally functioned as normal signaling functions can be ubiquitinated and degraded by molecules that have E3 ubiquitin ligase activity, such as Cbl-b, and thereby inhibit BCR signaling.^170–172^ In the future, drug therapy studies for autoimmune diseases and lymphomas can be based on the signaling regulators summarized in this article and to control and treat BCR signaling related diseases through siRNA and other means.^173^

This review focused on mature B cells containing mIgM, and discussed the regulatory mechanisms and regulators of the three main signaling pathways mediated by BCR. Compared with previous literature, this paper summarizes the positive and negative regulators that affect BCR signal transduction, and hopes to provide researchers with a macro view to further study the details of the BCR signaling pathway, or to conduct research on the related diseases through experimental control. However, cells are small, but all-embracing, and the regulatory mechanisms and regulators of signaling pathways are complex and trivial. Because of the short writing time and limited personal knowledge, this review is difficult to cover, and it would be a great honor for me if it was helpful.
